# Association of Early Atherosclerosis with Vascular Wall Shear Stress in Hypercholesterolemic Zebrafish

**DOI:** 10.1371/journal.pone.0142945

**Published:** 2015-11-12

**Authors:** Sang Joon Lee, Woorak Choi, Eunseok Seo, Eunseop Yeom

**Affiliations:** 1 Department of Mechanical Engineering, Pohang University of Science and Technology (POSTECH), San 31, Hyoja-dong, Pohang 790–784, Republic of Korea; 2 Department of New Biology, Daegu Gyeongbuk Institute of Science and Technology (DGIST), Dalseong, Daegu 711–873, Republic of Korea; University of Washington, UNITED STATES

## Abstract

Although atherosclerosis is a multifactorial disease, the role of hemodynamic information has become more important. Low and oscillating wall shear stress (WSS) that changes its direction is associated with the early stage of atherosclerosis. Several *in vitro and in vivo* models were proposed to reveal the relation between the WSS and the early atherosclerosis. However, these models possess technical limitations in mimicking real physiological conditions and monitoring the developmental course of the early atherosclerosis. In this study, a hypercholesterolaemic zebrafish model is proposed as a novel experimental model to resolve these limitations. Zebrafish larvae are optically transparent, which enables temporal observation of pathological variations under *in vivo* condition. WSS in blood vessels of 15 days post-fertilisation zebrafish was measured using a micro particle image velocimetry (PIV) technique, and spatial distribution of lipid deposition inside the model was quantitatively investigated after feeding high cholesterol diet for 10 days. Lipids were mainly deposited in blood vessel of low WSS. The oscillating WSS was not induced by the blood flows in zebrafish models. The present hypercholesterolaemic zebrafish would be used as a potentially useful model for *in vivo* study about the effects of low WSS in the early atherosclerosis.

## Introduction

Cardiovascular diseases (CVDs) are one of the major causes of mortality in western countries accounting for one in every three deaths in the US in 2009 [1]. Most CVDs are closely related to atherosclerosis. Atherosclerosis is usually initiated by an inflammatory process in the endothelial cells (ECs) of blood vessels [2]. The inflammatory process induces lipid-laden materials to deposit on arterial walls [3]. The deposit grows, forms fatty streaks and eventually closes off the affected artery after the formation of early-stage atherosclerosis.

The atherosclerotic deposits are predominantly observed in regions of curvature, bifurcation and branching of arterial vessels [4, 5]. Previous studies reported that hemodynamic conditions were disturbed in the regions, and the disturbed flow induces low or high oscillatory wall shear stress (WSS) on the ECs of arterial vessels [6–8]. The WSS is the skin frictional force per unit area acting on the wall, whose direction is parallel to local blood flow. The specific WSS condition is critical for the initiation and formation of early atherosclerosis. The processes encompass physiological changes in ECs, lipid accumulation and oxidation [9]. To reveal the pathology of WSS-induced early atherosclerosis, effects of WSS on morphological and physiological changes of ECs were investigated [10, 11]. Recently, chemical shear sensor systems and gene expressions of ECs were considered [12, 13]. However, the exact pathology is not fully revealed yet due to technological limitations encountered in experiments [6, 13].

The absence of suitable experimental model has been the main difficulty among the several obstacles encountered in revealing the pathology of WSS-induced early atherosclerosis. *In vitro* and *in vivo* models have been widely used to study the relations between WSS and early atherosclerosis [11, 14, 15]. In *in vitro* experiment, WSS over cultured EC monolayers was regulated by changing supplied flow rate [16, 17]. Estrada et al. [16] found that the size of cultured ECs under a constant flow condition was larger than that under the static condition. Ueki et al. [17] reported that the shear strain acting on ECs and nuclei of ECs was in proportion to the applied WSS. These results supported that the WSS can change the morphological and biophysical conditions of the ECs. The *In vitro* systems are useful for observing functional and morphological responses of the ECs according to WSS under precisely controlled experimental conditions. However, the *in vitro* condition is relatively different from the actual physiological environment of ECs in human blood vessels. This limitation was resolved by inducing early atherosclerosis in *in vivo* animal models such as pigs [18], rabbits [19] and mouse [20, 21]. High-fat diet was fed to the animals, or genes were manipulated to make these models. WSS is subsequently evaluated using several measurement techniques [22, 23]. The *in vivo* experiments with animal models have successfully observed lipid localisation in low or oscillatory WSS regions. However, atherosclerotic lesions in conventional animal models were commonly conducted with post mortem examinations [24–26]. These examinations have many problems in investigating the actual roles of WSS on the pathological time course of early atherosclerosis.

Zebrafish (*Danio rerio*) is a tropical freshwater fish which has been receiving considerable attention as a disease model for the study of embryological development [27, 28] or pathology of several circulatory vascular diseases [29]. Experimental studies using zebrafish have several advantages, such as ease of genetic manipulation and rapid generation time [30–32]. Moreover, an optical clarity of zebrafish enables a real-time monitoring of the developing pathologies. A research group recently developed a hypercholesterolaemic zebrafish model and investigated early atherosclerosis *in vivo* [33–36]. They observed vascular lipid accumulation, morphological and functional alteration of EC layer, recruitment of myeloid cells and lipid uptake by macrophages. However, the pathologic mechanism of early atherosclerosis related with haemodynamics was not fully elucidated.

In the present study, the hemodynamic characteristics of blood flows in the main blood vessels were measured, and focal distribution of lipid deposit in hypercholesterolaemic zebrafish models was investigated to demonstrate the feasibility of zebrafish as a WSS-induced early atherosclerosis model. Velocity field information was measured using a micro particle image velocimetry (μ-PIV) technique, and the focal lipid deposit was observed using a confocal microscope.

## Materials and Experiment Setup

### 2.1 Zebrafish and high-cholesterol diet

*Transgenic* (*fli1a*:*EGFP*) zebrafish with ECs expressing green fluorescent protein (GFP) was supplied by the Korea Zebrafish Organogenesis Mutant Bank (Daegu, Korea). Larvae of zebrafish were maintained at a room temperature of 28 ± 0.5°C under a 14 h:10 h light—dark cycle. A high-cholesterol diet was prepared by dissolving a normal feed (baby meal; Jail Feed Corporation, Korea) in a diethyl ether solution of cholesterol (Sigma Aldrich, USA) to a final content of 4% (w/w) cholesterol in the feed after evaporating the ether solution [33]. The feed was supplemented with 10 μg/g fluorescent cholesteryl ester analogue (cholesteryl BODIPY^®^ 542/563 C11; Molecular Probes) to investigate the lipid accumulation in vascular vessels of zebrafish. Zebrafish samples were fed twice in a day, starting from five days post fertilisation (dpf). All experimental procedures were approved by the Animal Care and Ethics Committee of POSTECH and the methods followed approved guidelines.

### 2.2 Experiment setup

A 15 dpf zebrafish was placed on 7% methylcellulose in a chamber (Chamlide TC; Live Cell Instrument, Korea) under anaesthesia by short exposure to 0.02% Tricaine. Two different measurement setups were utilized, depending on the region of interest. A microscope (Nikon, Japan) attached with a 20× objective lens (NA = 0.5) was employed to capture the flow images for the estimation of mean velocity in vessels. This relatively low magnification objective is suitable for capturing large areas of zebrafish at the same time. A modified fluorescence microscope (Zeiss Axiovert 200, Germany) with a 40× objective lens (NA = 0.6) was used to obtain velocity profiles and to observe the ECs of blood vessels. The zebrafish model was illuminated with a fluorescence excitation lamp (X-Cite 120 Q; Lumen Dynamics, Ontario, Canada) through a shift-free filter to identify GFP ECs. Sequential flow images were acquired with the illumination of halogen lamp at the centre plane of the vessel for 5 s, which corresponds to 12–15 cardiac cycles. Images of blood flows were obtained using a high-speed camera with 1 k × 1 k pixel resolution (Fastcam SA1.1; Photron, USA) at 125 frames to 500 frames per second, depending on the flow rate of each vessel. The effective pixel sizes for the two different measurement setups were 1 μm and 0.77 μm, respectively. All experiments were conducted at a room temperature of 28°C. All fishes were awakened in fresh water and returned to the fish tank immediately after each experiment.

### 2.3 μ-PIV

**Fig 1:**
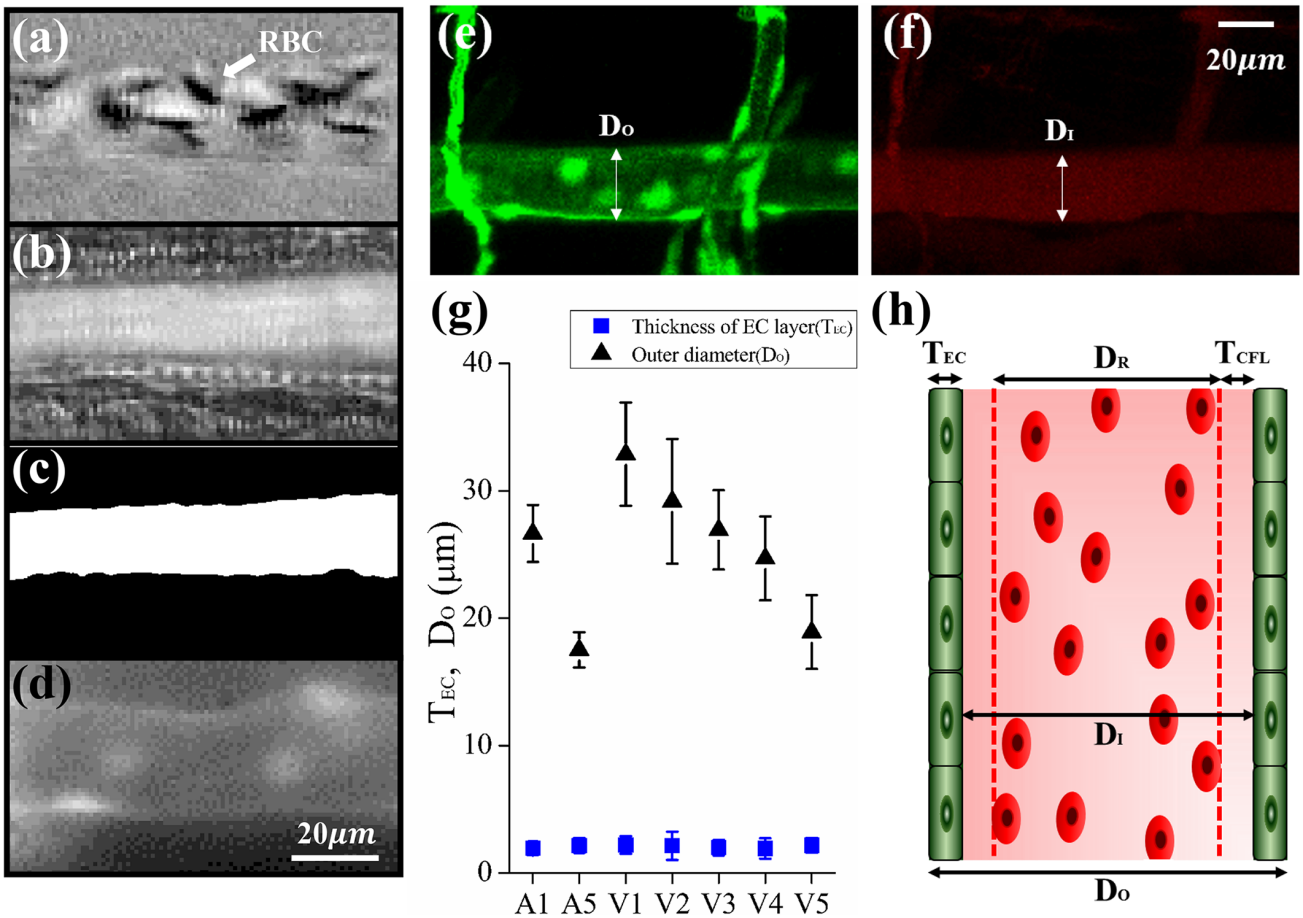
Identification of RBC-rich region and cell-free layer (CFL). (a) Bright field image of Artery 1 (A1) after subtraction of the background image. (b) Map of standard deviation (SD). (c) Binary image created by thresholding the SD map. (d) Fluorescent image of endothelial cells (ECs) on the vessel wall. Confocal microscopy image of (e) green fluorescent ECs on the vessel wall and (f) cholesteryl ester BODIPY 542/563 C11 circulating inside the blood vessel. The outer (*D*_*O*_) and inner (*D*_*I*_) diameters of the vessel were indicated by white arrows. (g) The *D*_*O*_ and thickness of EC layer (*T*_*EC*_) of each vessel were compared. (h) Terminologies should express the blood vessel, *T*_*CFL*_ (thickness of CFL) and *D*_*R*_ (diameter of RBC-rich region).

A FFT (fast Fourier transform)-based cross-correlation algorithm was applied to the flow images captured for PIV (particle image velocimetry) analysis. PIV technique determines the mean displacement of tracer particles in the consecutive interrogation windows by searching the location of a dominant peak in the corresponding cross-correlation plane [37]. RBCs (red blood cells) in blood were directly used as tracer particles. The sizes of each interrogation window for the two experimental setups were 32 × 8 pixels and 48 × 8 pixels. The physical dimensions of the two interrogation windows are similar (256 μm^2^, 228 μm^2^). Adjacent interrogation windows was 50% overlapped. A recursive correlation method with multiplication mode [38] was applied and Gaussian peak fit was employed to increase a sub-pixel measurement accuracy. To enhance signal-to-noise ratio in the cross-correlation, the background image obtained by averaging sequential images was subtracted from the original flow images (Fig 1a) [39]. All PIV analyses were performed using PIVview-2C (PIVTEC, GmbH) software. The velocity profiles in straight vessels were measured by cropping flow images and images of vessel wall into 100 × 60 pixels in size at the middle of each vessel. Unlike conventional PIV techniques, the volumetric illumination has been commonly employed in μ-PIV experiments. The depth of focus of an objective lens used in μ-PIV substituted for the role of the light sheet in conventional PIV. A halogen lamp was installed to give forward scattering. To obtain clear images of RBCs, a band-pass filter (550 ± 15nm) was installed in consideration of the absorption spectral peak of RBCs (~540nm) [40].

### 2.4 Confocal microscopy

A Leica confocal microscope (Leica Microscopy Systems, GMBH) with 10× and 40× objective lens (Leica Microscopy Systems, GMBH) was used to detect two fluorophores in zebrafish. Excitation laser was turned to 488 and 561 nm for GFP and BODIPY542/563 C11, respectively. The emission light from the two fluorophores was filtered using two different ranges of wavelength (GFP: 500–550 nm and BODIPY 542/563 C11: 565–605 nm). Each zebrafish sample was anaesthetised and mounted on a 7% methylcellulose. 3D confocal images reconstructed by using 2D section images were merged into a 2D image which was composed of maximum pixel intensity values along the depth direction. The merged images of GFP and BODIPY542/563 C11 (cholesteryl ester) were used to measure the outer (*D*_*O*_) and inner (*D*_*I*_) diameters of blood vessels, respectively (Fig 1). The images of BODIPY542/563 C11 were also utilized to analyse the quantity of lipid deposit. The acquired images were analysed and processed using LAS AF 2.7 software (Leica Microsystems Ltd. Germany). To increase the contrast between the images of circulating cholesteryl ester and deposited lipids, the pixel intensity values from 125 to 255 in the merged image (8bit) are rearranged to 0 to 255. When intensity values were smaller than 125, they were converted to 0. Most circulating cholesteryl esters were converted to 0, because they had relatively low intensity values. The yellow fluorescence from BODIPY 576/589 C11 was pseudo-color encoded to appear as red colour to clearly distinguish the green and yellow fluorescence in zebrafish, as depicted in Fig 1f.

## Analysis Methods

### 3.1 Vessel partition

**Fig 2:**
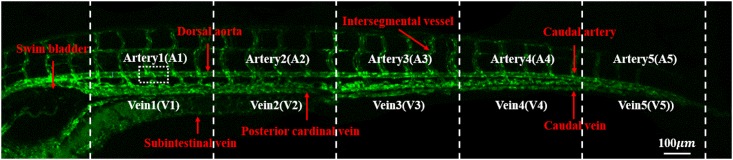
Angiogram of a zebrafish at approximately 15 days post-fertilisation (dpf). Vasculature was divided into 10 partitions by length: Arteries 1–5 (*A1–5*), Veins 1–5 (*V1–5*).

The average length of 15 dpf zebrafish models treated in this study was approximately 4.3±0.2 mm. As shown in the confocal microscope image of Fig 2, the main blood vessel was divided into 10 partitions, which were named from the first artery partition (*A1*) to the fifth vein partition (*V5*). These partitions were divided based on the length of the zebrafish sample, and the average vessel length of each partition was approximately 460 μm. The leftmost partitions, A1 and V1, were located at the right end of the swim bladder. Small vessels containing intersegmental vessels were not included in the region of interest in this study.

### 3.2 Identification of cell-free layer (CFL)

CFL was observed in the near-wall region of blood vessels of zebrafish because of the Fåhraeus—Lindqvist effect in small-scale vessels (Fig 1h) [41, 42]. Thus, this region should be identified to estimate WSS accurately. Fig 1 shows the mechanism of CFL identification in a vessel. The background image obtained by averaging the sequential images was subtracted from the original images to remove the static structures, including vessel walls. As shown in Fig 1a, the background-subtracted image clearly shows the RBC motion. RBC-rich regions could be easily distinguished by applying image processing techniques [43, 44]. In this study, the standard deviation (SD) map labelling the SD of image intensities in the subtracted images was adopted to depict the RBC-rich regions. As shown in Fig 1b, the pixels in the RBC-rich regions have large SD values because RBC movements induce large intensity fluctuations. An iterative selection thresholding method (ISTM) was applied to the SD map for conversion to binary images, in which the RBC-rich region was white and the remaining region of the image was black (Fig 1c) [45]. The threshold value in the ISTM was determined based on the median value between the mean intensity values of RBC-rich region and CFL. The binary images were used to determine the average diameter of the RBC region (*D*_*R*_) and utilised as a mask in PIV analysis. By analysing the GFP images of vessel wall captured by the fluorescence microscope (Fig 1d), the outer diameter of the blood vessel (*D*_*O*_) was obtained. However, the EC layers in the fluorescence images were not clearly distinct. The GFP images of EC layer and fluorescence images of cholesteryl BODIPY^®^ 576/589 C11 were obtained separately using confocal microscopy (Fig 1e and 1f). The D_O_ and D_I_ of blood vessel were measured based on the merged confocal images of green EC layer and red cholesteryl BODIPY^®^ 576/589 C11 circulating inside the vessel, respectively. The thickness of EC (*T*_*EC*_) was evaluated by subtracting *D*_*I*_ from *D*_*O*_ and then dividing by 2. The representative T_EC_ was obtained by averaging the data measured at the three points of each vessel (*A1*, *A5*, *V1*, *V2*, *V3*, *V4* and *V5*) in three zebrafish samples (Fig 1g).

Finally, the thickness of CFL (*T*_*CFL*_) was determined using the following equation:

$$T_{\text{CFL}}=\frac{D_{O}-2\times T_{\text{EC}}-D_{R}}{2}$$ (1)

All image processing procedures were conducted using a free-ware program (ImageJ, version 1.47).

### 3.3 WSS determination

The WSS in blood vessels was determined by assuming that plasma only contacts ECs because of the presence of CFL and the linear velocity profile of plasma in the gap between the edge of RBC-rich region and vessel wall. Previous studies utilised the same assumption to measure the WSS in arterioles (29.5–67.1 μm) of rat cremaster muscles [46] or the blood flows in rat skeletal muscle arterioles (21–115 μm) [47], with validation of measurement accuracy.

**Fig 3:**
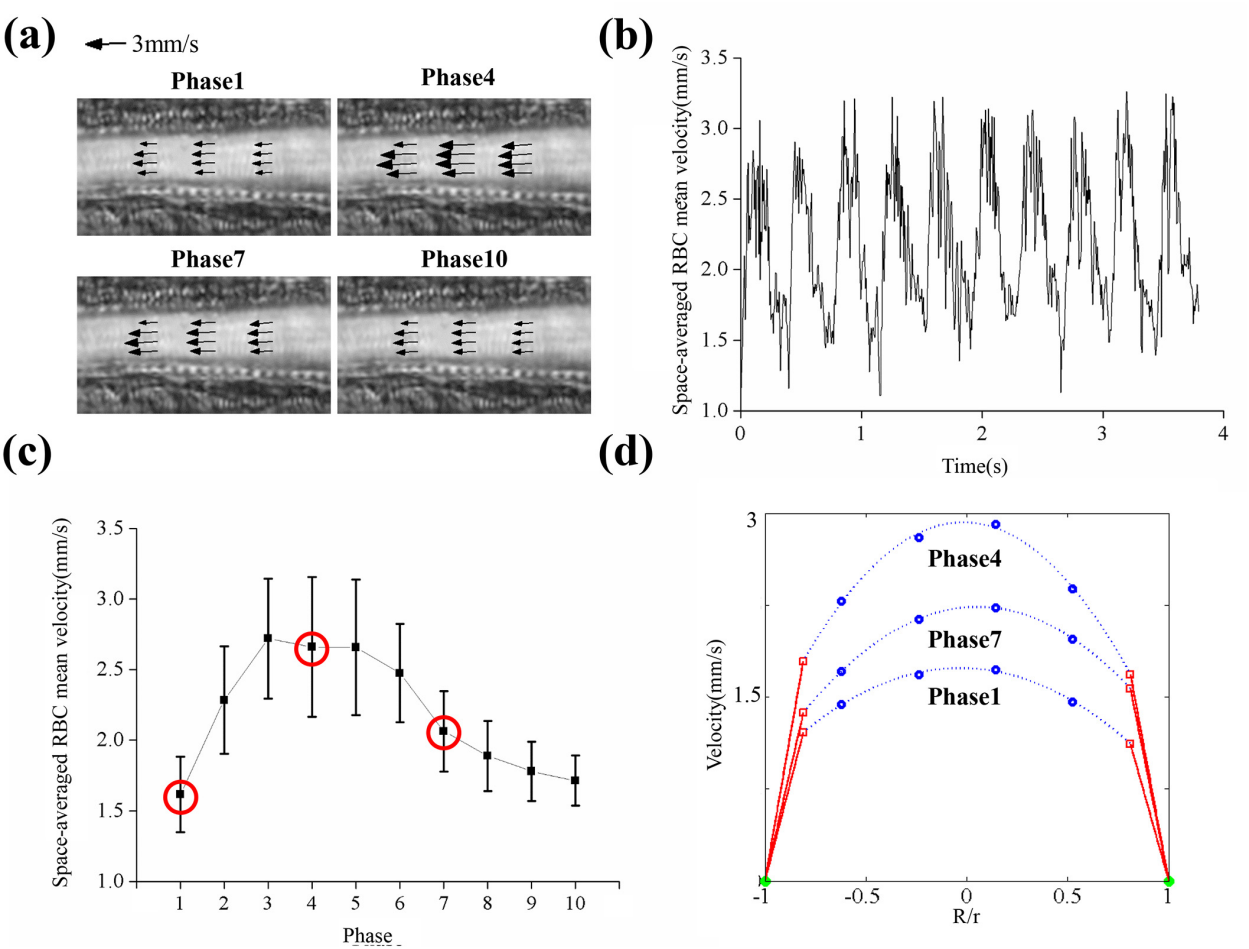
Measurement of wall shear stress in blood vessels. (a) Velocity vector fields in artery 1 (A1) at phase angles of 1, 4, 7 and 10 were overlaid with SD map. Each cardiac cycle was divided into 10 phases. (b) Temporal variation of Space-averaged RBC mean velocity (*V*_*SPACE*_). (c) Space-averaged RBC mean velocity (*V*_*SPACE*_) were averaged again in phase.(d) Velocity profiles at phases 1, 4 and 7. Red square dots: velocity magnitude of RBCs at the edge of RBC-rich region; blue dots: RBC velocity measured by μ-PIV technique; green dots: inner wall of blood vessel; blue dashed lines: fitting curve; red lines: velocity profile of plasma in CFL.

In the present study, WSS (*τ*_*w*_) was determined using the following equation:

$$\tau_{w}=\mu \frac{V_{\text{edge}}}{T_{\text{CFL}}}$$ (2)

where μ is the dynamic viscosity of plasma, and *V*_*edge*_ denotes the RBC velocity at the edge of RBC-rich region ((red dotted line in Fig 1h). Plasma is a Newtonian fluid, thus the plasma viscosity of zebrafish was assumed to be constant (1.2 cp) based on previous result that the viscosity of icefish plasma is similar to that of human plasma [48–51]. The value of *V*_*edge*_ was calculated by extrapolating RBC velocities measured by μ-PIV technique (blue circles in Fig 3d), with the help of second-order polynomial curve fitting.

We utilized 4 different average velocities in the present study (Table 1). The velocity fields at four different phases in A1 partition were overlaid with the SD map (Fig 3a). Fig 3b shows a temporal variation of the Space-averaged RBC mean velocity (*V*_*Space*_) for 10 cardiac cycles, with clearly shown pulsatile waveform. One cardiac cycle was divided into 10 phases, and phase averaging was conducted to enhance the measurement accuracy of WSS [52]. Fig 3c displays the phasic variation of the phase-averaged *V*_*Space*_. The portion of the deceleration phase was longer than that of the acceleration phase. The phase-averaged velocity profiles at three different phases were compared in Fig 3d. The WSS at each phase was determined by measuring the slope of red line at the corresponding phase.

**Table 1:** Terminology and definition of various average velocities.

Terminology	Definition (Averaging method)	Location
**RBC mean velocity**	Mean velocity of RBCs averaged along radial direction	
**Time-averaged RBC mean velocity (*V***_***Time***_ **)**	Mean velocity of RBCs averaged for 10 cardiac cycles	Fig 4
**Space-averaged RBC mean velocity (*V***_***Space***_ **)**	Mean velocity of RBCs space-averaged in each vessel	Figs 3 and 5
**Representative RBC velocity (*V***_***Rep***_ **)**	Average of all RBC velocities in each vessel for 10 cardiac cycles	Section 4.1

### 3.4 Statistical analysis

All data were expressed as mean value ± SD. Comparison between the two groups was conducted with a Student’s t test using Excel 2013 (Microsoft). An overall value of P ≤ 0.05 was considered statistically significant.

## Results

### 4.1 Haemodynamic flow characteristics

The heart rate of 15 dpf zebrafish treated in this study was approximately 165±28 beats per minute. Reynolds number (Re) was calculated based on the dynamic viscosity of whole blood (0.005 pa·s) [44], blood density *ρ* (1050 kg/m^−3^) and the representative RBC velocity (*V*_*Rep*_) of *A1* (≒1.245mm/s), *V5* (≒0.109mm/s). *Re* at vessels *A1* and *V5* were calculated as 0.005±0.002 and 0.0003±0.0001, respectively. Womersley number (*Wo*) is defined as follows:

$$\text{Wo = }\frac{D_{i}}{2}{\text{(}\frac{w\rho }{\mu_{\text{Blood}}}\text{)}}^{\frac{1}{2}}$$ (3)

where *ω* is the angular frequency of the cardiac cycle. The values of *Wo* at vessels *A1* and *V5* were 0.016±0.003 and 0.015±0.003, respectively. To validate our PIV analysis, we measured RBC mean velocity in the segment *A1* (dorsal aorta) of 3dpf (n = 3), 5dpf (n = 3) zebrafishes for which there are published data. The measured value is in reasonable agreement with the published results (3dpf (≒0.6mm/s) and 5dpf (≒1mm/s) zebrafish) (S1 Text) [53].

### 4.2 Pulsatile flow in main vessels

**Fig 4:**
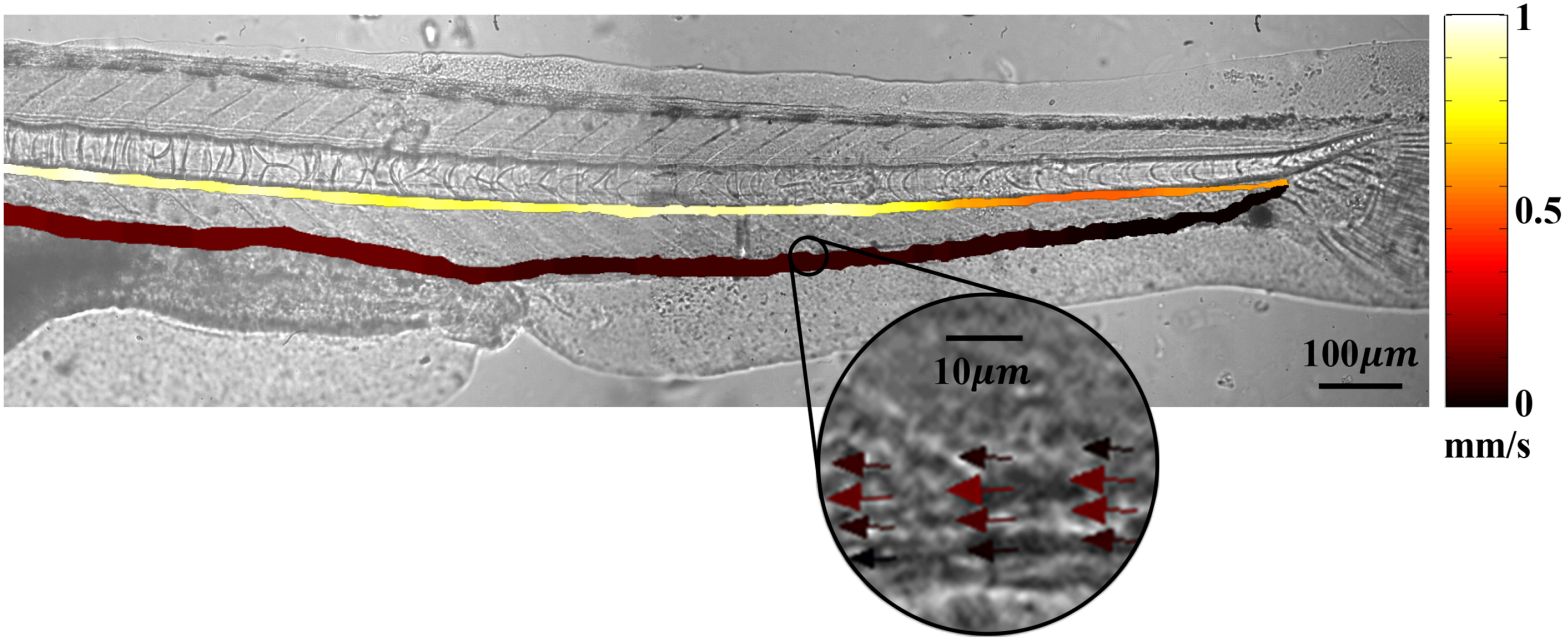
Color-coded Time-averaged RBC mean velocity (*V*_*TIME*_) in the main blood vessels. A selected region is enlarged to show the corresponding RBC velocity vectors.

The time-averaged RBC mean velocity (*V*_*Time*_) in the main vessels was represented with pseudocolouring (Fig 4). As a typical example, the vector field in the caudal vein (V4) was depicted using the corresponding pseudocolours. The upper and lower colour lines in Fig 4 represent the arterial and venous flows from the swim bladder to the tail, respectively. The binary image distinguishes the vasculature from the corresponding entire the bright field image (Fig 1C). An arterial flow moves towards the right side of the image, whereas the direction of a venous flow is the opposite. The *V*_*Time*_ in A1 was nearly 1 mm/s, and it gradually decreased flowing to the tail. By contrast, the minimum *V*_*Time*_ value near the tail was increased as the blood flow goes back to the heart.

**Fig 5:**
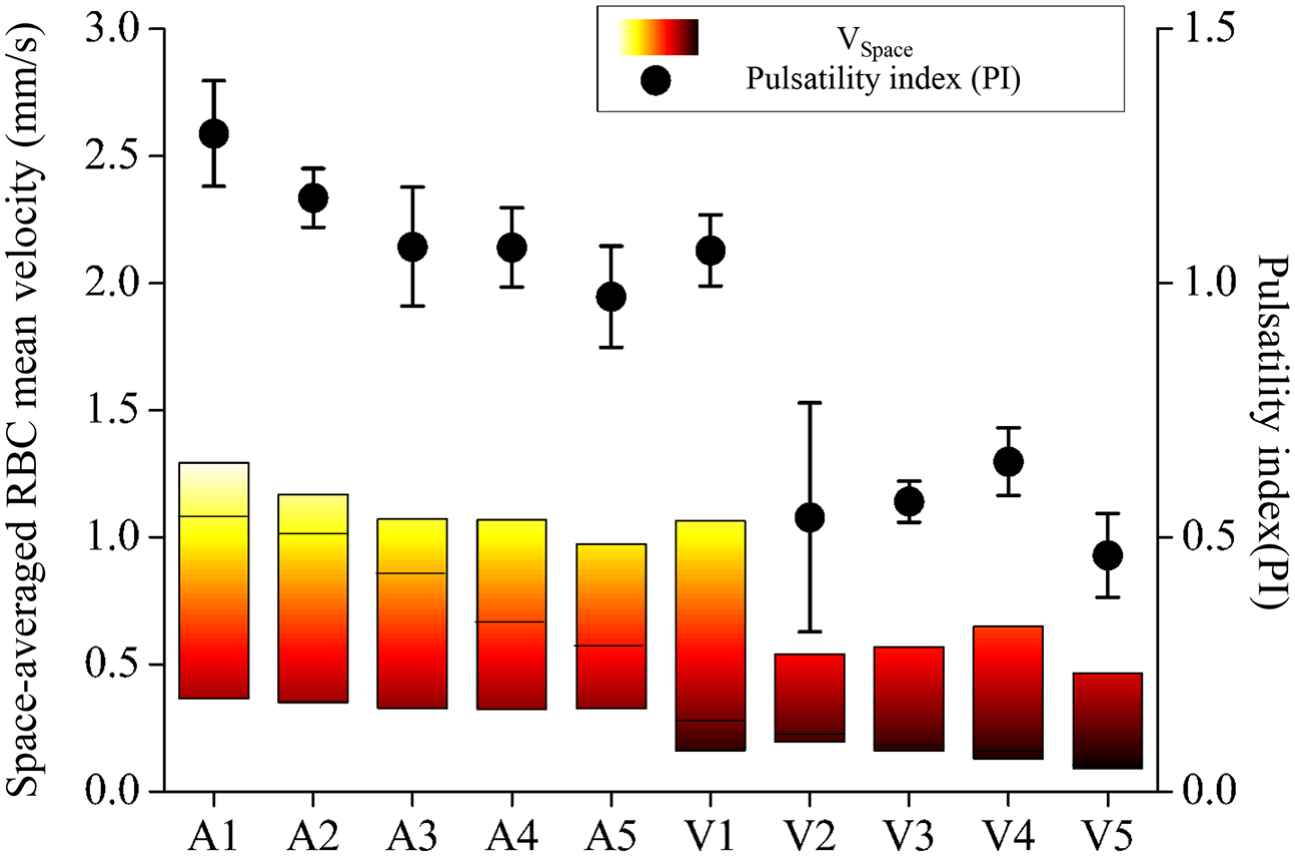
Variations of space-averaged RBC mean velocity (*V*_*Space*_), and pulsatility index (n = 3) along the main blood vessel. The horizontal line in each bar plot represents the median value.

Fig 5 compares the pulsatility of blood flow at each vessel segment (A1–A5, V1–V5). Colour bars represent the range of the RBC mean velocity during cardiac cycles. The range of *V*_*Space*_ was gradually reduced from A1 to V5. A pulsatility index (*PI*) was defined by the following equation to quantify the pulsatility of *V*_*Space*_ at each vessel.

$$\text{PI}=\frac{V_{\text{Max}}\text{-}\;V_{\text{Min}}}{V_{\text{Rep}}}$$ (4)

where *V*_*Max*_ and *V*_*Min*_ represent the maximum and minimum *V*_*Space*_ for the entire cycles, respectively. The variation trend of PI along the main vessel was generally similar to that of *V*_*Time*_, except for the first venous vessel V1. The pulsatile flow was unidirectional without any backflow in all the main vessels, as shown in Fig 5.

### 4.3 Comparison of WSS distribution

**Fig 6:**
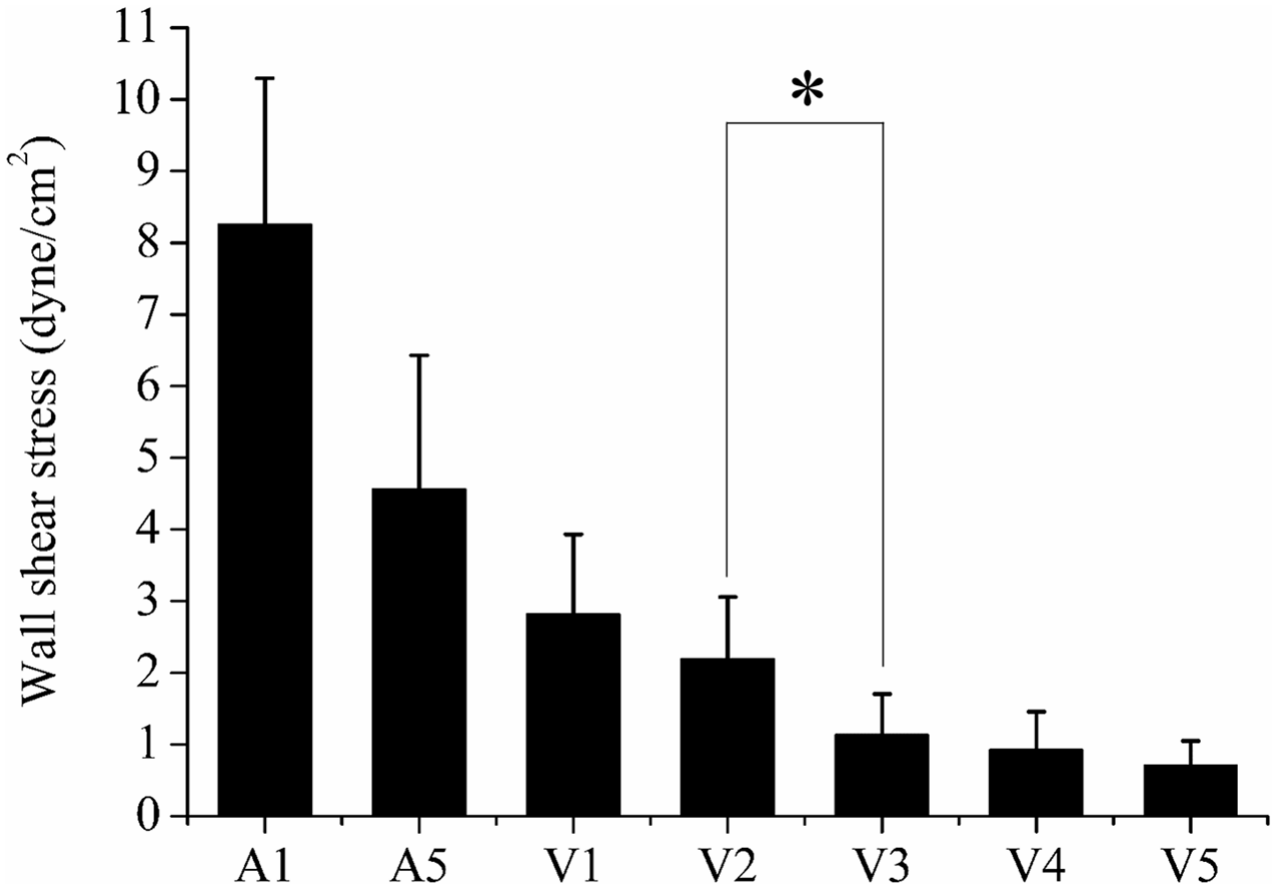
Time-averaged wall shear stress of pulsatile flow at seven vessel segments (n = 6) *P < 0.05.

A time-averaged WSS at each vessel partition was calculated by statistically averaging the variations of WSS for 10 phases. Fig 6 shows mean WSS of six zebrafish samples at seven vessel segments. The mean WSS at *A1* was 8.26 *dyne/cm* ^*2*^, whereas the values at *V3*, *V4* and *V5* were less than 1.2 *dyne/cm* ^*2*^.

### 4.4 Comparison of lipid accumulation

**Fig 7:**
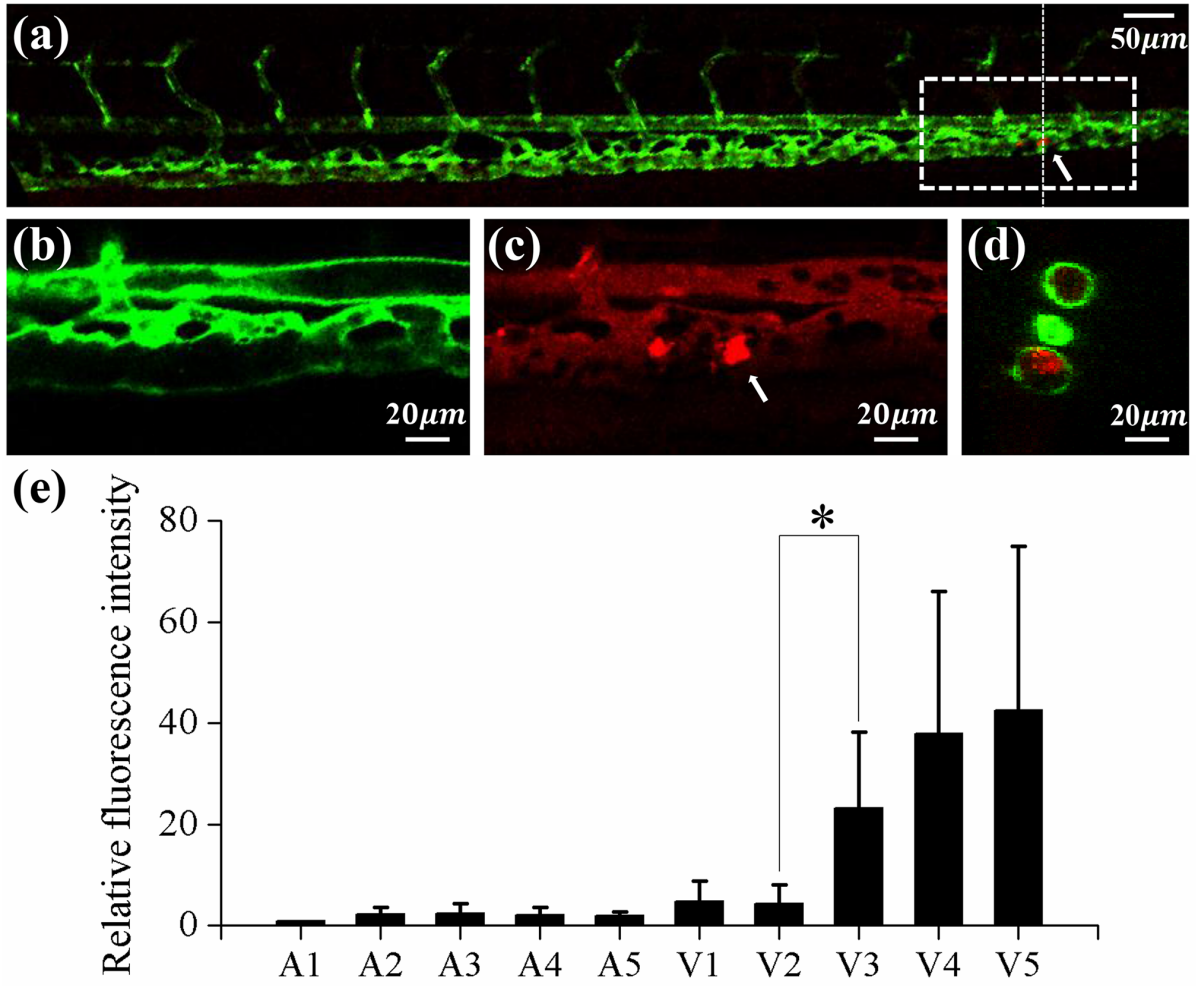
Lipid accumulation along the main blood vessel of 15 dpf zebrafish. (a) Typical confocal microscopy image of the caudal vasculature in a 15 dpf zebrafish. Bright fluorescent displays lipid deposit in the blood vessel. White arrows denote the location of lipid accumulation. ECs and fluorescent lipid image in the square dotted region were magnified in (b) and (c), respectively. (a)~(c) 2D sectional image, lateral view. (d) Cross sectional image at the vertical dotted line in (a). (e) Variation of relative fluorescence intensity of lipid in10 vessels. (n = 11) *P < 0.001.

Fig 7a shows the combined confocal 2D sectional image of ECs and cholesteryl ester. This image shows a lateral section at a center of the caudal vein (Fig 7a). A left side of the 2D sectional image and Fig 7b are somewhat blurry because the whole vessels were not placed on the same focal plane of the confocal microscopy. Several bright fluorescent spots were observed in the caudal vein near the tail (Fig 7a and 7c). Fig 7b and 7c show the magnified green and red images in a square box of Fig 7a. EC layers of the vessel wall (Fig 7b) and the lipid deposit on the vessel wall (Fig 7c) are clearly shown in the magnified images. Several black ellipses which indicate RBCs and circulating red cholesteryl ester are also shown in Fig 7c. A cross sectional image was acquired along the vertical dotted line marked in Fig 7a (Fig 7d) to observe the locations of the lipid deposits in the vessel wall more clearly.

Pixel intensities of red merged images (after applying digital image processing) were statistically averaged for each blood vessel to quantify the deposition of lipids. Then the averaged intensity at each vessel was divided by the value at the segment A1 for relative comparison among whole vessel segments (Fig 7e). Mean values of the relative pixel intensities at the segments *A1~A5*, *V1* and *V2* are smaller than 5, while the values at the segment *V3~V5* are larger than 23.

Control experiments were conducted by feeding normal diet without any supplementary cholesterol. Lipid deposits were not observed in blood vessels of the control group (S2 Text).

## Discussion

In this study, most lipids were deposited on veins near the tail where the measured WSS has low values. This result is in good agreement with the results obtained from human and other animal models [11, 54, 55]. Oshinski et al. evaluated WSS in aorta of eight healthy volunteers using MR phase velocity mapping technique [56]. They found that mean and peak WSS have small values in regions where early atherosclerosis is more likely to be formed. A similar relationship between low WSS and formation of plaque was reported in carotid artery [57], coronary artery [58], and blood vessels of animals [59, 60]. Although the deposit of lipids in the veins of zebrafish models is not well matched with the human atherosclerosis in arteries, it can be explained by the zebrafish vasculature in the early stage [33, 61, 62]. In the early stage, the main arteries and veins of zebrafish vasculature are directly connected, rather than interconnected through capillaries. This direct connection facilitates arterial blood flow to be supplied to the veins. A plenty of oxygen contained in the arterial flow has been known as one of important reasons of lipids deposit in arteries [63]. The specific condition was indirectly detected from the variation of the pulsatility index at each vessel of 15 dpf zebrafish models (Fig 5). The pulsatility index in the main artery decreases with approaching the tail, and the value is gradually recovered in the veins when the blood is returned to the heart. This implies that some portion of pulsatile blood flows in the main arteries is directly transferred to the main veins through intersegmental vessels (Fig 2).

The similar distribution of the value of *V*_*Rep*_ and WSS also can be explained by the same reason (Figs 4 and 6). *V*_*Rep*_ at the *V1* is smaller than that at the *A1* because the diameter of the *V1* is larger than the diameter of *A1*, and a portion of the blood flow in the main arteries returns to the heart through the subintestinal vein as shown in Fig 2 [61].

**Fig 8:**
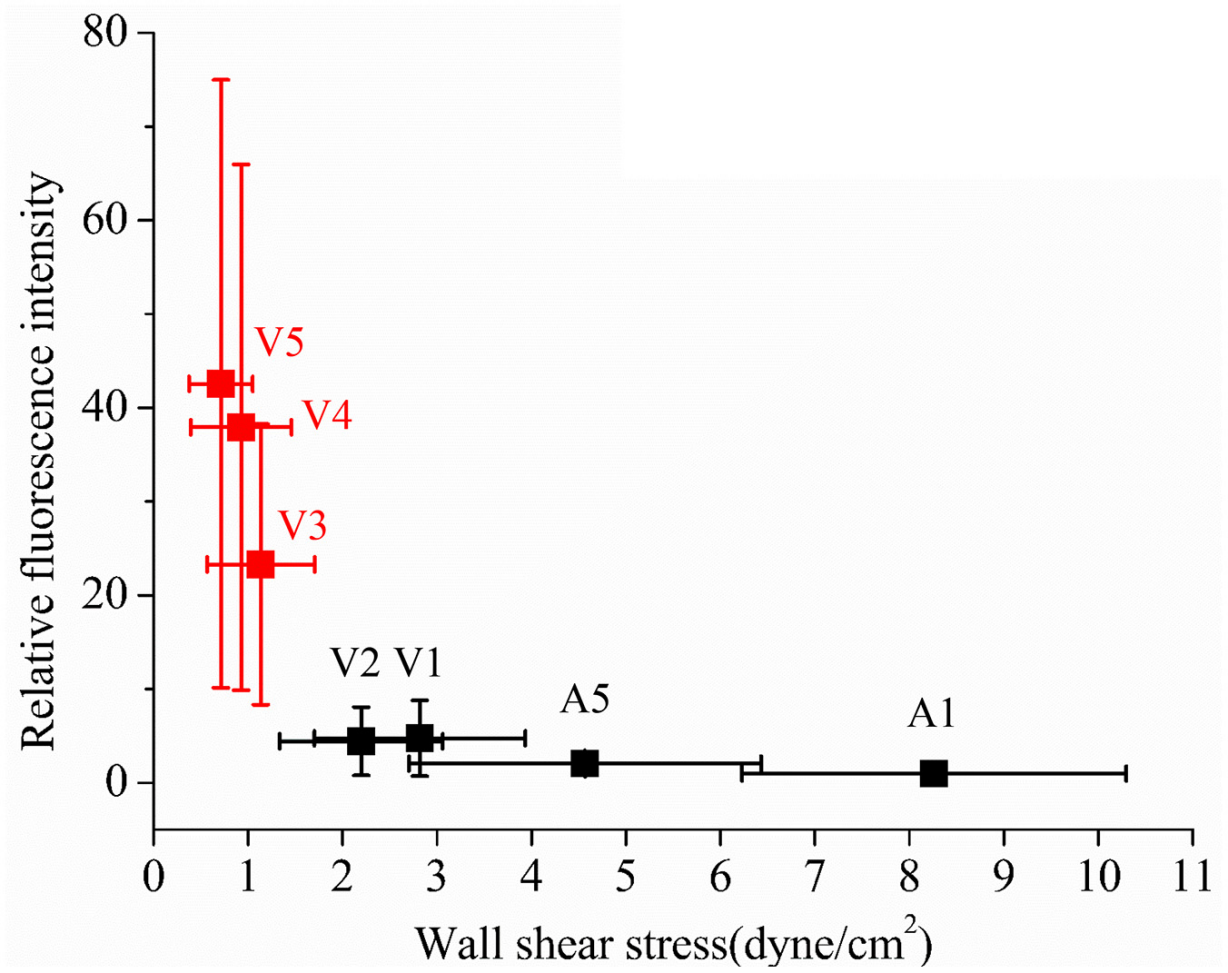
Relation between the relative fluorescence intensity and wall shear stress at seven vessels. The data at *V3–V5* are marked as red squares to distinguish the vessels where lipids were mostly deposited.

For a systematic investigation of the relation between WSS and early atherosclerosis in zebrafish, the variation of relative fluorescence intensity, which indicates the amount of lipid deposit, was examined according to the magnitude of WSS (Fig 8). The data at *V3–V5* were marked as red squares to distinguish the vessels where lipids were mostly deposited. The localization of lipid deposit was confirmed quantitatively by checking the confocal microscope images (Fig 7). The lipid deposit in a zebrafish can be interpreted as cholesteryl ester, free fatty acid, triglycerides and phospholipids as reported in a previous study. [33]. Stoletov et al. (2009) made a hypercholesterolemic zebrafish model using similar method to the present study, and observed that lipid deposit exhibits several inflammatory processes that occurred in the early atherosclerosis of human. Morphological and functional changes of ECs were observed in the location of lipid deposit. The symptom recruits myeloid cells, and transplanted macrophages uptake the lipids on the vessel of 15dpf zebrafish. They also observed deposition of lipids in the caudal vein. However, they did not reveal the reasons [33, 59]. Based on these results, localized deposition of lipids in low WSS regions can be considered as suitable evidence to suggest the zebrafish as a suitable disease model to study the WSS-induced early atherosclerosis.

The WSS distribution in each vessel was highly correlated with the RBC velocity because WSS was evaluated by *V*_*edge*_ calculated from the RBC velocity (Figs 4 and 6). The WSS measurements in segments *A2–A4* were not included to focus on low WSS condition. The WSS in the excluded vessels can be easily inferred to have relatively higher values than the low WSS region (*V3–V5*) from the values in *A1* and *A5* (Fig 6).

Atherosclerosis in human commonly occurs in large arteries, where the *Re* and *Wo* of blood flow reach 10^3^–10^4^ and 10, respectively [13]. The haemodynamic condition combined with complex vessel geometries leads to flow separation, recirculation and turbulent shear flow. These flow structures induce low or oscillatory WSS which direction is changed periodically. Previous studies reported that the oscillatory WSS condition is important with the low WSS condition in the outbreak of early atherosclerosis [64, 65]. In the present study, the blood flow in 15 dpf zebrafish exhibited low *Re* (< 0.01) and *Wo* (< 0.02) in the main vessels. Turbulent flow or the complex flow structures were not observed in 15 dpf zebrafish because of low *Re*. The low *Wo* caused the velocity profile along the radial direction to always remain unidirectional without a flow reversal as shown in Figs 3a and 4 [66].

Zebrafish is suitable for studying the effects of low WSS on the outbreak of early atherosclerosis, and it is not suitable for studying the effects of oscillatory WSS. The zebrafish model has potential to disclose unknown physiology about low WSS-induced early atherosclerosis. Its unique strength to observe the temporal progression of the disease with detailed haemodynamic information in *in vivo* condition would help to overcome the limitation of previous experimental models. In addition, the WSS-induced change of ECs observed in the zebrafish model can be used as a new index for early diagnosis of atherosclerosis. The results imply that zebrafish model can be utilized for drug discovery to block the pathology of early atherosclerosis. The *in vivo* monitoring of the effects of drugs at low prices can be unique strength [67].

Although the relationship between low WSS and lipid deposit was clearly shown, the changes of ECs, mid-process between low WSS condition and lipid deposition, was not demonstrated in the present study. Study on the effect of low WSS on the functional features of ECs is required as a future work. The WSS-induced lipid deposit focused in the present study occurs in the early stage of atherosclerosis. An examination on the growth of atherosclerosis beyond the early atherosclerosis in zebrafish models would be another interesting research topic.

## Conclusion

WSS was measured in the main blood vessels of 15dpf zebrafish with micro-PIV technique, and the lipid deposits at every blood vessels were compared quantitatively by using the confocal microscope images. A clear relation between low WSS condition and the lipids deposit was demonstrated. It implies that zebrafish can be utilized as a suitable animal model for the research about the low WSS-induced early atherosclerosis. The unique strengths of zebrafish model would be helpful to reveal the pathology of early atherosclerosis and to search effective diagnostic methods or therapeutic drugs for the disease.

## Supporting Information

S1 Text RBC mean velocity in the segment A1 (dorsal aorta) of 3dpf (n = 3), 5dpf (n = 3) zebrafishes. (DOCX)

S2 Text Confocal microscope images of two control zebrafish models. (DOCX)
