# Supplementary material for: Association of Early Atherosclerosis with Vascular Wall Shear Stress in Hypercholesterolemic Zebrafish
Source: PLoS One. 2015 Nov 12;10(11):e0142945. doi: 10.1371/journal.pone.0142945 (PMC4643039; doi:10.1371/journal.pone.0142945)
Supplement: S1 Text — (DOCX) [file pone.0142945.s001.docx]

**Supporting information**

**S 1. Validation of PIV results**

To validate our PIV analysis, we measured RBC mean velocity in the segment *A1* (dorsal aorta) of 3dpf (n=3), 5dpf (n=3) zebrafishes for which there are published data. The measured value is in reasonable agreement with the published results (3dpf (≒0.6mm/s) and 5dpf (≒1mm/s) zebrafish) (Watkins et al. 2012).


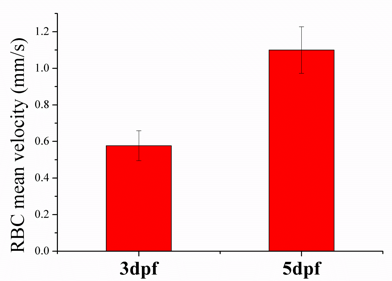


Fig. S 1 RBC mean velocity in the segment A1 (dorsal aorta) of 3dpf (n=3), 5dpf (n=3) zebrafishes

**Reference**

53. Watkins SC, Maniar S, Mosher M, Roman BL, Tsang M, St Croix CM. High resolution imaging of vascular function in zebrafish. PloS one. 2012;7(8).
