# Supplementary material for: Association of Early Atherosclerosis with Vascular Wall Shear Stress in Hypercholesterolemic Zebrafish
Source: PLoS One. 2015 Nov 12;10(11):e0142945. doi: 10.1371/journal.pone.0142945 (PMC4643039; doi:10.1371/journal.pone.0142945)
Supplement: S2 Text — (DOCX) [file pone.0142945.s002.docx]

**Supporting information**

**S 2. Confocal microscope images of two control zebrafish models**

Control experiments using zebrafish fed normal diet for 10 days were conducted (the same amount of fluorescent cholesteryl ester without additional cholesterol). As shown in the figure below, deposition of lipids on blood vessels was not observed in the confocal microscope images of the control group.


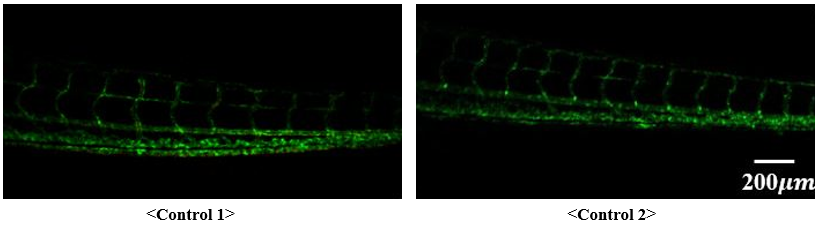


Fig. S 2 Confocal microscope images of two control zebrafish models.
